# Triple A syndrome presenting as complicated hereditary spastic paraplegia

**DOI:** 10.1002/mgg3.492

**Published:** 2018-10-31

**Authors:** Etienne Leveille, Hernan D. Gonorazky, Marie‐France Rioux, Lili‐Naz Hazrati, Jennifer A. Ruskey, Amanda Carnevale, Dan Spiegelman, Alexandre Dionne‐Laporte, Guy A. Rouleau, Grace Yoon, Ziv Gan‐Or

**Affiliations:** ^1^ Faculty of Medicine McGill University Montréal Québec Canada; ^2^ Division of Neurology, Department of Pediatrics, The Hospital for Sick Children University of Toronto Toronto Ontario Canada; ^3^ Department of Neurology Université de Sherbrooke Sherbrooke Québec Canada; ^4^ Department of Laboratory Medicine and Pathobiology, The Hospital for Sick Children University of Toronto Toronto Ontario Canada; ^5^ Montreal Neurological Institute McGill University Montreal Quebec Canada; ^6^ Department of Neurology and Neurosurgery McGill University Montréal Québec Canada; ^7^ Division of Clinical and Metabolic Genetics, Department of Pediatrics, The Hospital for Sick Children University of Toronto Toronto Ontario Canada; ^8^ Department of Human Genetics McGill University Montréal Québec Canada

**Keywords:** AAAS, hereditary spastic paraplegia, triple A syndrome

## Abstract

**Background:**

Hereditary spastic paraplegia (HSP) is a group of rare disorders characterized by spastic paraparesis and other symptoms. Often, other diseases can mimic HSP, which may delay diagnosis and treatment.

**Methods:**

Whole exome sequencing was performed in families with clinically suspected HSP without a genetic diagnosis.

**Results:**

We report three patients from two families who presented with lower limb spasticity, muscular atrophy, and other neurological symptoms, who were clinically diagnosed with complicated HSP. Whole exome sequencing revealed bi‐allelic *AAAS* nonsense mutations; one individual was homozygous for the p.(Arg478*) mutation, and two siblings were homozygous for the p.(Arg286*) mutation, leading to the diagnosis of triple A syndrome. This rare syndrome is typically characterized by a triad of symptoms: achalasia, adrenal insufficiency, and alacrima, and is often accompanied by other neurological abnormalities.

**Conclusions:**

Our findings suggest that triple A syndrome should be suspected in complicated HSP patients without a known genetic cause, especially if at least one of the main triad of triple A syndrome symptoms is present.

## 1 INTRODUCTION

Hereditary spastic paraplegias (HSPs) are a group of rare neurological disorders with a prevalence of 2–10/100,000 individuals (Ruano, Melo, Silva, & Coutinho, 2014; Salinas, Proukakis, Crosby, & Warner, 2008). HSP is a genetically and clinically heterogeneous disease characterized by axonal degeneration of the descending corticospinal tract and ascending sensory fibers (Deluca, Ebers, & Esiri, 2004; Lo Giudice, Lombardi, Santorelli, Kawarai, & Orlacchio, 2014). Pure HSP symptoms include progressive spasticity and weakness of the lower limbs, neurogenic bladder disturbance, deep tendon hyperreflexia, and extensor plantar response, while complicated HSP also includes other neurological abnormalities such as ataxia, epilepsy, intellectual disability, dementia, and deafness (Gan‐Or et al., 2016; Salinas et al., 2008). There are more than 70 genes or genetic loci that are known or suspected to be involved in HSP, and numerous other disorders may present with spasticity and mimic HSP. Identifying these mimicking disorders is important as they should be considered in the differential diagnosis of HSP.

Triple A syndrome, also known as achalasia‐addisonianism‐alacrima (triple A) syndrome (OMIM 231550), is a rare autosomal recessive disorder, caused by mutations in the *AAAS* gene (Handschug et al., 2001; Tullio‐Pelet et al., 2000; Weber et al., 1996). Symptoms typically manifest during childhood with alacrima. Adrenal insufficiency is common during the first or second decades of life and can provoke lethal adrenal crises. Achalasia typically follows during the first or second decade of life (Kilicli, Acibucu, Senel, & Dokmetas, 2012; Prpic, Huebner, Persic, Handschug, & Pavletic, 2003; Tibussek et al., 2018). Neurological symptoms are progressive and can present at any age with various manifestations (Dixit, Chow, & Sarkar, 2011; Dumic et al., 2011).

Various neurological abnormalities in triple A syndrome may overlap with those present in upper and/or lower motor neuron diseases such as amyotrophic lateral sclerosis (ALS) or HSP. Herein, we report three patients from two families recruited to CanHSP, a Canada‐wide network for HSP research (Chrestian et al., 2017), who were initially diagnosed with HSP based on their clinical presentation and later confirmed to have triple A syndrome.

## 2 MATERIALS AND METHODS

### 2.1 Population

Hereditary spastic paraplegia patients and their family members were recruited through CanHSP, a Canada‐wide consortium for HSP research, as previously described (Chrestian et al., 2017). The two consanguineous families discussed in the current paper were recruited in Toronto (family A) and Montreal (family B), and HSP was diagnosed based on previously published criteria by neurologists specialized in motor neuron diseases (Gasser et al., 2010). All patients and family members provided written informed consent to participate in this study, and the study protocol was approved by the institutional review boards.

### 2.2 Genetic analysis

DNA was extracted from whole blood using a standard salting out protocol, and whole exome sequencing (WES) was performed using the Agilent SureSelect Human All Exon V4 Kit according to the manufacturer's (Agilent Technologies) instructions at the Montreal Neurological Institute, Montreal, Canada. Further details on the WES and validations can be found in the Appendix S1.

## 3 RESULTS

**Figure 1:**
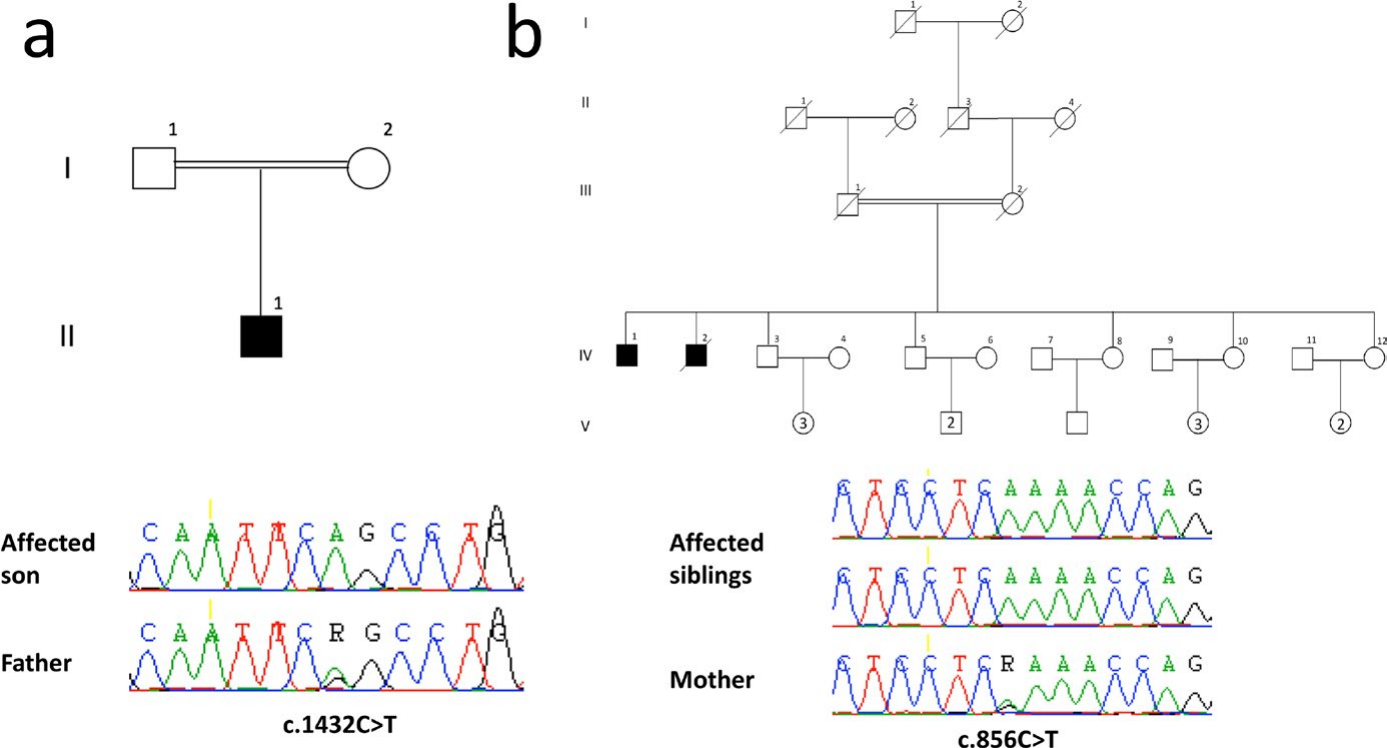
Pedigrees and mutations found in the current study. (a) Pedigree of family A with a patient with triple A syndrome, initially diagnosed with hereditary spastic paraplegia (HSP), who is homozygous for the c.1432C>T (p.R478*) nonsense mutation. The chromatogram is of the reverse (antisense) sequence. (b) Pedigree of family B with two patients with triple A syndrome, also initially diagnosed with HSP, who are homozygous for the c.856C>T (p.R286*) nonsense mutation. The chromatogram is of the reverse (antisense) sequence

**Figure 2:**
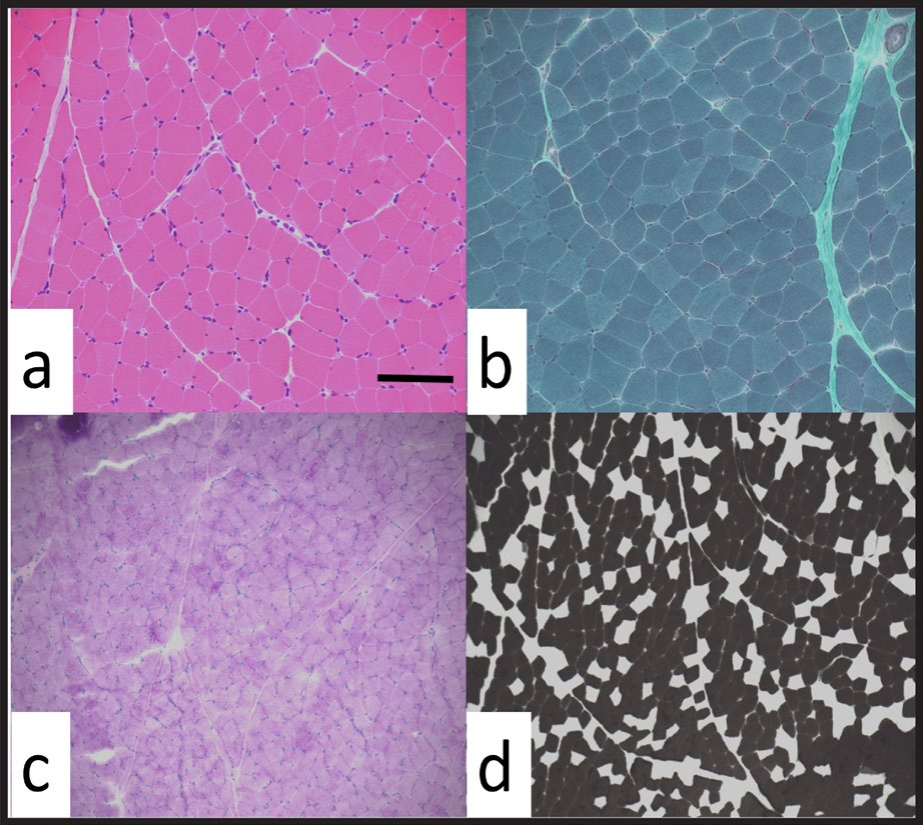
Photomicrographs of histology and histochemical features of the muscle biopsy from patient 1. (a) H&E stained section shows minimal variation in muscle fiber size. (b) Modified Gomori trichrome and (c) PAS, both showing normal pattern of staining. (d) ATPase at 4.3 pH shows predominance of type I fibers. Scale bar in a and b represent 200 and 400 μm in c and d

The two families were consanguineous (Figure 1); one of Guyanese origin (Family A) and one of French‐Canadian origin (family B). In all three patients, a clinical diagnosis of complicated HSP was given during follow‐up of the patients. Interestingly, in all three patients a specific diagnosis of Troyer syndrome (OMIM 275900, SPG20) was hypothesized during the course of the disease, but genetic tests for *SPART* (OMIM 607111, *SPG20*) were negative. Detailed clinical description of the three patients and the clinical, genetic, and biochemical tests can be found in the online Appendix S1. As these patients were enrolled in CanHSP (Chrestian et al., 2017), WES was performed, and the three patients were subsequently diagnosed with triple A syndrome. In patient 1 (Family A), WES identified a homozygous mutation in the *AAAS* gene (NM_015665.5), c.1432C>T leading to p.(Arg478*). Both parents were confirmed as heterozygous carriers with Sanger sequencing (Figure 1). Muscle biopsy revealed predominance of type 1 fibers (80.4% type 1% vs. 19.6% type 2), which gives the focal impression of fiber type grouping (Figure 2, additional data in the Appendix S1). In patients 2 and 3 (Family B), homozygous c.856C>T *AAAS* mutations leading to an early stop p.(Arg286*) were identified. Table 1 details the main clinical characteristics of the three patients related to triple A syndrome and compares them with data from other triple A syndrome patients with the same mutations from the literature.

**Table 1:** Features of triple A syndrome and comparison with previously reported patients with the p.R478* and p. R286* mutations

	Patient 1 (current study)	Patient A[Link]	Patient B[Link]	Patient C[Link]	Patient D[Link]	Patient E[Link]	Patient F[Link]	Patient 2 (current study)	Patient 3 (current study)	Patient G[Link]	Patient H[Link]	Métis‐Canadian family[Link]
Mutation	p.R478*	p.R478*	p.R478*	p.R478*	p.R478*	p.R478*/p.Q387*	p.R478*	p.R286*	p.R286*	p.R286*/c.1368_1372delGCTCA	p.R286*	p.R286*
Age at onset (years)	2	0	0	2.5	<5	0	1	2	Unknown	0	4	Unknown
Age (years)	14	3.5	8.5	3.5	33	15.6	5	70	Died at 47	12	4	2–29
Adrenal insufficiency	+	+	+	+	+	+	+	−	Unknown	+	+	8/8
Achalasia	−	+	+	−	+	+	−	+	+	+	+	8/8
Alacrimia	−	+	+	+	+	+	+	+	Unknown	+	+	8/8
Intellectual disability	−	+	+	−	+	−	−	−	−	+	−	5/8
Muscle weakness	+	−	−	−	+	+	−	+	+	−	−	2/8
Hyperreflexia	+	−	−	−	+	−	−	+	Unknown	+	−	Unknown
Ataxia	−	−	−	−	−	−	−	+	+	+	−	0/8
Optic atrophy	−	−	−	−	+	−	Unknown	+	Unknown	−	Unknown	0/8
Sensory impairment	−	−	−	−	−	−	Unknown	−	−	−	−	Unknown
Palmar and plantar hyperkeratosis	+	+	+	+	+	+	+	−	−	+	Unknown	3/8

Data taken from references (Brooks et al., 2004; Goizet et al., 2002; Handschug et al., 2001; Kallabi et al., 2016; Milenkovic et al., 2010; Moore et al., 1991; Singh et al., 2018; Yuksel et al., 2004).

Compound heterozygous individuals.

Numbers indicated represent the number of individual with the features within the families (8 individuals total).

## 4 DISCUSSION

Many triple A syndrome patients present with only one or two elements of the achalasia–adrenal insufficiency–alacrima triad, in addition to other neurological manifestations (Gazarian, Cowell, Bonney, & Grigor, 1995; Houlden et al., 2002). Patients with an incomplete triad and predominant neurological features such as cerebellar ataxia, upper and lower motor neuron signs, and muscle atrophy may receive a diagnosis of juvenile ALS, Charcot–Marie–Tooth disease, or other neurologic disorders (Dumic et al., 2011; Ismail, Tulliot‐Pelet, Mohsen, & Al‐Saleh, 2006; Karle et al., 2013; Reimann et al., 2017; Strauss et al., 2008). In this report, the three patients were suspected to have complicated HSP, as spastic paraplegia with amyotrophy was their main presenting symptom, at initial presentation and throughout the disease course. Therefore, triple A syndrome should be suspected in HSP patients without a genetic diagnosis, especially if one of the triad symptoms is present, and the *AAAS* gene should be included in panel screening for HSP. As detailed in Appendix S1, we generated a panel of 695 genes (Table S1) which are known or suspected to be involved in HSP, or may have similar features that may mimic HSP, and this panel of genes can be screened in HSP patients who undergo WES.

The phenotype of triple A syndrome is variable even in individuals with the same genotype. (Brooks et al., 2005; Houlden et al., 2002; Kinjo et al., 2004; Prpic et al., 2003) In six previously described cases of triple A syndrome with the p.(Arg478*) *AAAS* mutation (Table 1), four had achalasia and one patient had recurrent vomiting episodes, although achalasia could not be confirmed. In patient 1, upper gastrointestinal studies demonstrated absence of achalasia. In addition, patient 1 had progressive spasticity of the lower extremities, while four of the six other cases with the p.(Arg478*) mutation had no motor symptoms. However, all seven patients had adrenal insufficiency (Goizet et al., 2002; Milenkovic et al., 2010; Singh et al., 2018; Yuksel et al., 2004). A total of 10 individuals from three families with p.(Arg286*) mutations in *AAAS* have previously been reported (Table 1) (Brooks et al., 2004; Handschug et al., 2001; Kallabi et al., 2016; Moore, Couch, Perry, Shuckett, & Winter, 1991), all with achalasia, alacrimia, and adrenal insufficiency. Alacrimia was reported by patient 2 but could not be confirmed in patient 3. Adrenal insufficiency was not reported in both siblings, although hypogonadism was suspected in patient 3 (Appendix S1). Other neurological features are not common in previously described individuals with p.(Arg286*) mutations, while they are predominant in patients 2 and 3. These genotype–phenotype differences may suggest that there are other factors, genetic or environmental, which may affect the presentation of triple A symptoms.

It has been suggested that the triple A triad should be expanded to include autonomic and other neurological abnormalities and that diagnosis should be suspected in individuals with progressive neurological problems and at least one element of the triad (Gazarian et al., 1995; Houlden et al., 2002). Our current findings support this suggestion, and it is possible that earlier diagnosis could have been achieved, especially in family B, had these been the recommendations. Of note, early genetic testing and diagnosis can prevent lethal adrenal crises, as patients are at risk for cortisol deficiency (Grant et al., 1993; Patt et al., 2017). Therefore, we reiterate the need to consider triple A syndrome in patients diagnosed with complicated HSP or other neurological disorders with at least one of the classical triple A syndrome symptoms.

## CONFLICT OF INTEREST

All authors report no conflict of interests.
