# Supplementary material for: Triple A syndrome presenting as complicated hereditary spastic paraplegia
Source: Mol Genet Genomic Med. 2018 Oct 31;6(6):1134–9. doi: 10.1002/mgg3.492 (PMC6305671; doi:10.1002/mgg3.492)
Supplement: Supplementary file 2 [file MGG3-6-1134-s002.docx]

**Supplementary material**

**Allgrove syndrome presenting as complicated hereditary spastic paraplegia**

**Running title:** Allgrove syndrome presenting as HSP

Etienne Leveille,^1,*,^ Hernan D. Gonorazky,^2,*,^ Marie-France Rioux,^3,^ Lili-Naz Hazrati,^4,^ Jennifer A. Ruskey,^5,6,^ Amanda Carnevale,^7,^ Dan Spiegelman,^5,6,^ Alexandre Dionne-Laporte^,5,6,^ Guy A. Rouleau,^5,6,8,^ Grace Yoon,^2,7,^ Ziv Gan-Or,^5,6,8^.

Affiliations:

1) Faculty of Medicine, McGill University, Montréal, Québec, Canada

2) Division of Neurology, Department of Pediatrics, The Hospital for Sick Children, University of Toronto, Toronto, Ontario, Canada

3) Department of Neurology, Université de Sherbrooke, Québec, Canada

4) Department of laboratory medicine and pathobiology, The Hospital for Sick Children, University of Toronto, Toronto, Ontario, Canada

5) Montreal Neurological Institute, McGill University, Montreal, Quebec, Canada

6) Department of Neurology and Neurosurgery, McGill University, Montréal, Québec, Canada

7) Division of Clinical and Metabolic Genetics, Department of Paediatrics, The Hospital for Sick Children, Toronto, Ontario, Canada

8) Department of Human Genetics, McGill University, Montréal, Québec, Canada

Corresponding authors:

Grace Yoon, MD, FRCP(C)

Divisions of Neurology and Clinical and Metabolic Genetics

The Hospital for Sick Children

University of Toronto

555 University Avenue

Toronto, Ontario M5G 1X8 CANADA

phone: 416-813-6389

fax: 416-813-5345

email: grace.yoon@utoronto.ca

Ziv Gan-Or

Department of Neurology & Neurosurgery,

Department of Human Genetics,

Montreal Neurological Institute, McGill University

1033 Pine Avenue, West,

Ludmer Pavilion, room 327

Montreal, QC, H3A 1A1

Phone: +1-514-398-5845

e-mail: ziv.gan-or@mcgill.ca

* Equal contribution.

**Material and methods – full description**

*Population*

HSP patients and their family members were recruited through CanHSP as previously described ^1^. The two families discussed in the current paper were recruited in Toronto (family A) and Montreal (family B), and HSP was diagnosed based on previously published criteria by neurologists specialized in motor neuron diseases ^2^. All patients and family members signed informed consent forms, and the study protocol was approved by the institutional review boards.

*Genetic analysis*

DNA was extracted from whole blood using a standard salting out protocol, and whole exome sequencing (WES) was performed using the Agilent SureSelect Human All Exon V4 Kit according to the manufacturer’s (Agilent Technologies) instructions at the Montreal Neurological Institute, Montreal, Canada. Filtering was applied to select mutations with a frequency lower than 0.005 on the Exome Aggregation Consortium (ExAC) database. Non-synonymous, frameshift and stop-gain variants that segregated with the disease were filtered in, and the online prediction and conservation tools SIFT, PolyPhen-2, MutationTaster, PhyloP and GERP++ were used to estimate the potential effects of the mutations. We generated a list of all genes known or suspected to be involved in HSP, as well as genes that are associated with diseases that can mimic HSP or may have spasticity as one of their symptoms (a total of 695 genes, Supplementary Table 1), and cross-examined the filtered-in variants with this list. Validation and further segregation of the suspected pathogenic mutations was performed using Sanger sequencing (Applied Biosystems, 3730x DNA Analyzer technology). DNA was amplified using forward primer 5’-TCCCCTACTGTACCCCACTG-3’ and reverse primer 5’-CCCAGCTCATCACTTTCCAT-3’ in family A and forward primer 5’-CCTCCCCAGTGTCTGTGAAT-3’ and reverse primer 5’-GATGATGGGGTGCTTGAGTT-3’ in family B, and the chromatograms were examined using the Genalys 3.3b software.

**Results**

***Clinical description of patients and genetic findings***

*Family A - Patient 1*

The patient was born through uncomplicated vaginal delivery after a full-term, unremarkable pregnancy to consanguineous parents of Guyanese background (Figure 1). His initial developmental milestones were normal until two years of age, when he developed progressive gross motor difficulties due to progressive lower extremity spasticity and toe walking. Electromyography (EMG) and nerve conduction study (NCVs) performed at age 9 years revealed decreased amplitude of the compound muscle action potentials (CMAPs), and he was given a diagnosis of complex HSP. Brain and spine MRI at 9 years was reported as normal. At 11 years he presented with hyperpigmentation of his tongue, severe spasticity and generalized muscle atrophy. This later progressed to include atrophy and fasciculation of the tongue, and generalized, severe muscle cramps. A repeat EMG and NCVs performed at 12 years revealed reduced amplitude of the CMAPs with normal sensory nerve action potentials (SNAPs). EMG revealed normal insertional activity, however at rest there was evidence of fibrillations and acute positive sharp waves (1+) on minimal contraction. The motor unit potentials were polyphasic with normal duration and increased amplitudes. On maximal contraction there was reduced recruitment. These results were compatible with a lower motor neuron involvement. Somatosensory Evoked Potentials and Visual Evoked Potentials revealed delayed cortical responses. A diagnosis of HSP was given, with the clinicians suspecting Troyer syndrome (SPG20) or Silver syndrome (SPG17), yet genetic tests for mutations and deletions in these genes were negative.

At 14 years old, after enrollment in CanHSP, the patient received a diagnosis of adrenal insufficiency, in addition to lower extremity progressive weakness and spasticity, toe walking, language delay and chronic diarrhea. On physical exam, the extraocular movement and pupils were normal. The tongue was atrophic and fasciculations were present. The remainder of the cranial nerves were normal. Muscle bulk was diminished throughout, especially the distal muscles of the upper limbs. Strength was decreased for deltoids, interossei, lumbricals, iliopsoas, gluteus maximus, tibialis anterior and extensor hallucis longus (4/5 symmetric). He had hyperreflexia with an abnormal jaw jerk. Bilateral extensor plantar response with bilateral clonus and Hoffman’s sign were present. Sensory examination was normal. He had hyperpigmentation of his gums, tongue, palmar creases and axilla.

Microarray, genetic testing for Juvenile ALS and metabolic tests (urine organic acids and plasma amino acids, lactate, carnitine, acylcarnitine, pipecolic acid, phytanic acid, very long fatty acids, CK levels and vitamin B12) were normal. Upper GI series did not reveal any signs of achalasia. Cortisol level was <10 (low), ACTH stimulation test revealed a peak level of 19 (low**)**, and renin was normal. Muscle biopsy revealed predominance of type 1 fiber (80.4% type 1 vs 19.6% type 2), which gives the focal impression of fiber type grouping (Figure 2). ATPase reactions showed the fiber sizes to be within normal limits; the mean fiber diameter of type 1 fibers was 32.57 microns and 31.07 microns for type 2 fibers. Mitochondrial, acid phosphatase, Glycogen and lipid studies were all normal. Ultrastructural examination of the biopsy did not reveal any specific changes.

WES analysis identified homozygous mutations in the *AAAS* gene (NM_015665.5f, p.(Arg478*), c.1432C>T), and both parents were confirmed as carriers with Sanger sequencing (Figure 1).

*Family B - patient 2*

The patient was born after a full-term pregnancy as the second child of healthy parents of French-Canadian descent. Both parents are from the same geographical region, but consanguinity was not known. He had nuchal cord at birth, but with no adverse sequelae. Psychomotor development was normal until he was two years old, when he presented with significant dysphagia that required a temporary gastrostomy and intubation, which led to vocal chords paralysis. Since then, the patient has been having trouble feeding and is on a soft foods diet. He was referred to endocrinology at 13 years old for achalasia with megaesophagus with a family history (of his brother, patient 3) of achalasia, failure to thrive, hypogonadism and erectile dysfunction. Cortisol levels were normal. Motor function was within normal range during adolescence.

At age 28, the patient presented with a clinical picture consistent with a progressive motor syndrome with upper and lower motor neuron involvement. Spinal muscular atrophy and Kennedy syndrome were excluded by molecular testing, and other diagnoses such as Spastic paraplegia 20, also known as Troyer syndrome, and familial juvenile amyotrophic lateral sclerosis were suggested. Through adulthood, the patient was followed up mainly for his dysphagia and dysphonia, with slowly progressing motor symptoms, and received a diagnosis of HSP without known genetic cause.

Evaluation at 64 years of age showed that the patient had spastic gait and ataxia. He had no sensory or cognitive complaint, and was able to walk unaided. On physical examination, the patient was emaciated and had predominantly distal severe muscular atrophy. There was mild weakness (4/5) in the muscles of the hands and the feet. Extra-ocular movements and visual fields were normal. Tongue was atrophic and fasciculation were present. Hoffman sign was positive bilaterally, and plantar reflex was equivocal. Electrophysiologic studies and clinical evaluation suggested a mixed sensory-motor polyneuropathy with axonal predominance. Extensive neuropathy workup was done and the patient gave consent for genetic studies.

Initially, whole exome sequencing did not identify pathogenic mutations, but a recent re-analysis of the data identified a homozygous c.856C>T mutation in the *AAAS* gene leading to an early stop p.(Arg286*) mutation, found also in the patient’s brother (Patient 3). The mutation was validated with Sanger sequencing. His mother was confirmed as a heterozygous carrier, a sample from the father was not available for analysis. The patient is now 70 years of age, and in addition to prior symptoms, he complains of recurrent syncope or alteration of mental status. An EEG was performed and was normal. The patient has no history of epilepsy, no incontinence, no myoclonus, no seizures and no tongue biting. The patient is still able to walk without walking aids, but has weakness and decreased manual dexterity in his hands. He reports having occasional paresthesia in the hands. Gait is spastic with normal proprioception. The patient is followed for optic nerve atrophy and reports that he has never produced tears.

*Family B – Patient 3*

This patient is the younger brother of patient 2 (Figure 1). Information on this patient is limited as he was last seen in 2003 and died three years later at the age of 47. His clinical presentation was similar to his brother’s, with a more severe upper motor neuron syndrome and atrophy that was more distal than proximal. He also had prominent dysarthria and a pseudobulbar affect. Hypogonadism was suspected at a young age due to failure to thrive, yet no additional details are available on the workup. Hoffman sign was positive bilaterally. Spinal muscular atrophy, Kennedy syndrome and very long chain fatty acid tests were negative. Spastic paraplegia 20 (Troyer syndrome) was hypothesized, yet the genetic screening for *SPART* mutations was negative. The patient died with a diagnosis of a motor neuron disease, either ALS or HSP. Recent analysis of whole exome sequencing identified that this patient also carried a homozygous c.856C>T (p.(Arg286*)) mutation.

**References**

1. Chrestian N, Dupre N, Gan-Or Z, et al. Clinical and genetic study of hereditary spastic paraplegia in Canada. *Neurol Genet.* 2017;3(1):e122.

2. Gasser T, Finsterer J, Baets J, et al. EFNS guidelines on the molecular diagnosis of ataxias and spastic paraplegias. *Eur J Neurol.* 2010;17(2):179-188.
